# EDPNet: An Encoding–Decoding Network with Pyramidal Representation for Semantic Image Segmentation

**DOI:** 10.3390/s23063205

**Published:** 2023-03-17

**Authors:** Dong Chen, Xianghong Li, Fan Hu, P. Takis Mathiopoulos, Shaoning Di, Mingming Sui, Jiju Peethambaran

**Affiliations:** 1 College of Civil Engineering, Nanjing Forestry University, Nanjing 210037, China; 2 Department of Informatics and Telecommunications, National and Kapodistrian University of Athens, 15784 Athens, Greece; 3 School of Geosciences and Info Physics, Central South University, Changsha 410083, China; 4 Department of Mathematics and Computing Science, Saint Mary’s University, Halifax, NS B3P 2M6, Canada

**Keywords:** semantic segmentation, semantic parsing, pyramidal representation, encoder–decoder network, convolution neural network

## Abstract

This paper proposes an encoding–decoding network with a pyramidal representation module, which will be referred to as EDPNet, and is designed for efficient semantic image segmentation. On the one hand, during the encoding process of the proposed EDPNet, the enhancement of the Xception network, i.e., Xception+ is employed as a backbone to learn the discriminative feature maps. The obtained discriminative features are then fed into the pyramidal representation module, from which the context-augmented features are learned and optimized by leveraging a multi-level feature representation and aggregation process. On the other hand, during the image restoration decoding process, the encoded semantic-rich features are progressively recovered with the assistance of a simplified skip connection mechanism, which performs channel concatenation between high-level encoded features with rich semantic information and low-level features with spatial detail information. The proposed hybrid representation employing the proposed encoding–decoding and pyramidal structures has a global-aware perception and captures fine-grained contours of various geographical objects very well with high computational efficiency. The performance of the proposed EDPNet has been compared against PSPNet, DeepLabv3, and U-Net, employing four benchmark datasets, namely eTRIMS, Cityscapes, PASCAL VOC2012, and CamVid. EDPNet acquired the highest accuracy of 83.6% and 73.8% mIoUs on eTRIMS and PASCAL VOC2012 datasets, while its accuracy on the other two datasets was comparable to that of PSPNet, DeepLabv3, and U-Net models. EDPNet achieved the highest efficiency among the compared models on all datasets.

## 1. Introduction

Semantic image segmentation is an essential task in various computer vision fields. Fox example, in medical image processing [1,2], semantic segmentation is utilized in tumor border extraction and organ/tissue measurement, which helps doctors to make scientific judgments quickly and efficiently. In agricultural fields [3,4,5], semantic segmentation of remote sensing images helps to map and monitor land use and land cover (LULC) changes for sustainable land development, planning, and management. In the autonomous driving system applications [6,7], vehicles need the semantic information of the surrounding scene to assist their understanding and perception of complex traffic situations. In this way, the vehicles can identify lane markings, traffic signs, traffic lights, and other generic objects, thereby achieving real-time lane-level positioning and navigation in normal driving conditions. However, semantic image segmentation suffers from occlusions, low contrast, unfavorable perspectives, and lacking depth information. These factors can make automatic image understanding difficult. In addition, scenes captured in the physical world are diverse with different classes and varying greatly in size. The number of samples per class in an image can also vary significantly, leading classifiers to be biased towards the dominant classes, such as the background or other majority classes. These destructive effects can result in under- and/or over- segmentation issues. To deal with such problems, numerous methods have been proposed over the past few decades to enable differentiation of different semantic objects within an image. According to the different degrees of self-learning, the existing semantic segmentation algorithms can be roughly divided into three categories: traditional segmentation, machine learning-based segmentation, and deep learning-based segmentation.

## 2. Related Work

### 2.1. Traditional Segmentation

The traditional semantic segmentation method can be further classified into “threshold-based” semantic segmentation, “edge-based” semantic segmentation, and “region-based” semantic segmentation based on differences in their algorithmic implementation.

Threshold-based Semantic Segmentation: This class of methods utilizes the gray value difference between the object and the background to segment foreground/background pixels from images. It is simple, computationally efficient, and easy to implement. However, the biggest challenge is choosing the most appropriate thresholds to perceive single or multiple objects from the images. Generally, trial and error setting, statistical analysis [8], and maximum between-class variance [9] strategies are frequently used to classify pixels into several semantic categories. Instead of using one fixed threshold for the entire image, the adaptive thresholds with variable values are used for different sections of an image. More specifically, the input images are first segmented into multiple patches, and the threshold values for each are calculated. In this way, the threshold-based method not only considers the intensity of each pixel but also takes the spatial features of the entire image into account. Threshold-based semantic segmentation is widely used for parsing simple scene images but is very challenging for parsing complex and diverse scenes due to the noise shading effect and illumination changes, thereby leading to fuzzy boundaries without sharp features.

Edge-based Semantic Segmentation: Such method operates by detecting discontinuities in an image according to the differences in texture, contrast, grey level, spectral bands, saturation, and other properties. Once the edge pixels with high probability are determined, the image scene can be segmented into different semantic patches. The confidence of scene edge is often based on first-order derivative operators such as the Roberts [10], Prewitt [11] and Sobel [12] as well as second-order derivatives such as Laplacian of Gaussian (LOG) [13] and Difference of Gaussian (DOG) [14]. Another important classic detection method is the Canny edge detector [15], which optimizes the trade-off between edge detection and localization. Edge-based semantic segmentation is straightforward and easy to implement. However, it faces significant challenges when processing complex image scenes with rich details because of over- and/or under-segmentation problems. Moreover, this method is vulnerable to noise due to the delicate nature of edge structures [16].

Region-based Semantic Segmentation: This method segments the entire image into different patches with similar properties by propagating from predefined seed pixels or regions. The most commonly used algorithm for classification is the region-growing method [17], which begins with a series of seed pixels and then performs a propagation operation by merging adjacent pixels according to a particular criterion. To enhance the robustness and stability of this method, a region splitting and merging strategy [18] is often employed. In particular, it first splits the entire image into multiple superpixels with similar properties and then iteratively merges the neighborhood superpixels that have similar similarities. Other variants of region-growing methods such as watershed [19], probability density clustering [20], connected-component clustering [21], and density-based spatial clustering [22] are extensively used for semantic image parsing under various scenarios. In summary, region-based semantic segmentation is robust with a high anti-noise ability. However, the success of this algorithm heavily relies on the chosen seeds, clustering criteria, and propagation strategies.

### 2.2. Machine Learning-Based Segmentation

Machine learning methods often transform the segmentation problem into an optimization problem by constructing an objective function. The objective optimization helps to cluster the pixels in the feature space to achieve pixel-level semantic parsing. For example, the conventional classical *K*-means clustering [23], fuzzy *C*-means clustering [24], and expectation maximization (EM) clustering [25] optimize the objective function to achieve high intra-class similarity and low inter-class similarity in the feature space. However, these methods require a specific number of semantic categories, and using an incorrect number of semantic classes can result in over- and/or under-segmentation of images. Other methods, such as the Hough transform [26] and the random sample consensus (RANSAC) [27] algorithm, construct objective functions from a statistical perspective to realize the semantic image segmentation through a voting mechanism, but in general the computational and space complexity of semantic segmentation is higher than others. AdaBoost [28] and Random Forest [29] construct an objective optimization function from the perspective of information gain to finalize semantic image scene parsing, although the segmentation results are often limited by training accuracy. In summary, for the construction of objective functions for unsupervised machine learning, the accuracy mainly depends on the feasibility of the objective function and the difficulty of solving its solution. For supervised semantic image parsing, the accuracy is mainly limited by the chosen classifier and feature representation. Even though excellent feature operators such as SIFT [30] and SURF [31] exist, they are challenging to extract discriminative features from complex image scenes.

### 2.3. Deep Learning-Based Segmentation

The existing deep learning-based semantic image segmentation methods are committed to solving the following two problems. (a) The multi-scale target segmentation problem: this problem is closely related to the image scene complexity. That is, objects with different classes exhibit different sizes. In some cases, even though the objects have the same semantic properties, they still vary in shape and size. Therefore, deep learning-based networks need to capture the semantics of the image at different scales to recognize the targets that are too large and/or too small. (b) The precise boundary prediction problem: this problem arises from the network structure, which contains many convolution and pooling layers to increase the receptive field and decrease the computational complexity. At the same time, it also reduces the output resolution of the network. For the end-to-end task of semantic image segmentation, if we cannot effectively recover the lost spatial/location information, the local details of object boundary will be weakened, thus leading to lower semantic accuracy.

For the two problems mentioned above, we summarize the deep semantic segmentation networks into two categories, namely “deep pyramidal architecture” and “auto-encoder-decoder architecture”. We will review the relevant works from these two categories to explore the network’s mechanisms and highlight the various approaches which can be applicable for semantic image segmentation.

Deep pyramidal networks: Such networks utilize pyramid architectures with different resolutions. The most straightforward way to construct this architecture is through downsampling using convolution and pooling operations, which are inherent in CNNs. These pyramidal feature maps are solely predicted and fused to achieve accurate visual recognition. For instance, the SSD [32] network employs six different size feature maps to detect objects with various scales. Low-level feature maps are used to predict small targets, while high-level feature maps predict large targets. The predictions obtained on all the feature maps are fused for the regression and classification of the target bounding boxes. FPN [33] creates a feature pyramid with high-level semantics for detecting objects at different scales. The pyramidal feature hierarchy consisting of feature maps at several scales is first computed by three bottom-up downsamplings. After that, the coarse resolution feature maps are upsampled by three top-down upsampling. The upsampled features in each scale are merged with features of the same level from bottom-up downsampling through lateral connections. The constructed feature pyramid with rich semantics at all levels can help to recognize multi-scale objects accurately. The PANet [34], taking FPN as a baseline, adds another bottom-up aggregation network to fuse pyramidal features with the lowermost feature for instance segmentation. It enhances the entire feature hierarchy with accurate low-level localization information by shortening the propagation path between lower and topmost features. Other FPN variations use NAS-FPN [35] and BiFPN [36] to obtain enhanced multi-scale features. The HED-UNet [37] produces a feature pyramid consisting of six different resolutions by five times of upsampling in the decoder to predict the coastline segmentation of each layer’s feature map. The predictions at different levels are then compared with the ground truth at multi-resolution to form deep supervision to improve the training efficiency and generalization ability. The SPP-net [38] identifies all regions corresponding to the region proposals in feature maps, then splits them into 1, 4, and 16 patches. Max-pooling is conducted on these multi-level spatial patches, and the pooling results are concatenated to generate a feature vector with a fixed size. This mechanism makes it possible to process input images of arbitrary sizes or scales during training, thereby increasing scale invariance and reducing over-fitting in classification and object detection tasks. YOLOv5 (https://github.com/ultralytics/yolov5, accessed on 19 February 2023) constructs feature pyramid pooling by extending the original SPP module to SPPF (spatial pyramid pooling fast) module [38], which concatenates multiple small pooling kernels to replace them with large ones in SPP. SPPF significantly reduces the computational complexity of object detection. In contrast, Fast R-CNN [39] and Faster R-CNN [40] only adopt a pyramidal level RoI pooling module as a particular case of the SPP module to convert any RoIs’ features into a small feature map with a fixed length. These fixed-size feature maps are then fed into a fully convolutional layer for bounding-box regression and object classification. The PSPNet [41] uses a pyramid parsing module (PPM) to encode the feature maps of images at multiple scales. Specifically, PPM contains four different pyramid scale features. The coarsest level is generated by global pooling, while other levels divide the input features into different sub-regions and generate the pooled feature representation regarding different sub-regions. The pooled features at different levels are upsampled to the same size, followed by concatenation to form the global pyramidal features.

There exist also other pyramidal variations which have been proposed to encode the feature maps in different ways. One frequently used technique is Atrous convolution with different atrous rates, which can generate feature maps with varying resolutions. For example, DeepLabv2 [42] proposes the atrous spatial pyramid pooling (ASPP) module, which uses multiple branches of filters with different atrous rates to capture multi-scale feature maps. Another approach is the spatial pyramid dilated convolution operation with different dilation rates, as used in ESPNet [43], which represents feature maps at various resolutions while reducing the number of network parameters for a lightweight network. Short connection can also be employed to fuse feature maps at different resolutions. A series of publications along this line demonstrates the effectiveness of this mechanism. For example, ResNet [44] uses a shortcut connection to skip two successive layers and form residual learning blocks, enabling the training of very deep neural networks. DenseNet [45] combines any layers between inputs and outputs through skip connection to make the feature propagation easier. Finally, HRNet [46] directly assembles multi-resolution parallel subnetworks and achieves multi-scale feature fusion by exchanging information across these subnetworks. As such, it simultaneously considers global semantics and local appearance details.

Auto-encoder-decoder networks: These networks are designed to map images from an input domain to an output domain in two stages. Firstly the encoder takes an input image and aggregates features at multiple levels to generate high-dimensional features. Secondly, the decoder predicts the semantic segmentation mask from the high-dimensional feature space. The encoder–decoder network achieves a good balance between semantic prediction and boundary localization during the task of semantic image segmentation. For example, FCN [47] uses pooling and convolution operations to reduce feature map size and increase the number of channels, while the decoder adopted deconvolution for upsampling. To alleviate the information losses which occured during encoding and decoding, FCN combines fine layers and coarse layers to make local prediction accurate. To better retain the local boundary features, DeconvNet [48] combines pooling and deconvolution operations to strengthen the accurate, detailed representation. SegNet [49] records the pooling indices beforehand, and during decoding, it restores the feature maps to their original size according to these indices. U-Net [50], as a classical segmentation network specifically designed for medical images, consists of an encoder and a decoder. In the encoding stage, the input images are downsampled to achieve feature aggregation by using consecutive convolutional operations and max pooling. At the stage of decoding, up-convolution is used to align the sampled feature map of the corresponding layer from the encoder. The feature maps of the same depth in the encoder–decoder layers are fused directly using a skip connection to supplement the information of the decoder. Based on the classic U-Net, most diverse variants have been proposed by enhancements of skip connection, backbone design, bottleneck, and rich representation or by combining the classic U-Net with different types of Transformer or probabilistic extensions [1]. These U-Net extensions make the feature map representation discriminative in various visual recognition tasks. In RefineNet [51], the output of a pre-trained ResNet is downsampled and upsampled four times at the encoder and decoder part, respectively, to yield the feature map of the original input size. After a series of convolution, pooling, and fusion operations, RefineNet combines the feature map generated by the encoder layer with the output of the decoder layer in the previous stage, making the fusion of multi-scale features more in-depth. EncNet [52] introduces contextual information for context encoding, which helps to capture the semantic context of scenes and selectively highlight class-dependent features. ExFuse [53] modifies the channel parameters of the backbone network at the encoder stage and places the auxiliary supervision in the shallow layer of the encoder to assess the quality of the shallow features. Then, this approach conducts convolution and interpolation operations on the high-level feature maps, thus obtaining the same number of channels and size as the low-level feature maps. By introducing the semantics into low-level features and integrating the details into high-level features, image semantic segmentation accuracy is significantly improved.

### 2.4. Unresolved Issues

Pyramidal structure networks have a strong ability in identifying small-sized features but perform poorly in aggregating contour features for semantic image segmentation. This is because the augmented features derived from the pyramidal structures have a much smaller resolution by multi-scale hierarchical learning. However, pyramidal structure networks can capture multi-scale semantic representation due to their inherent deep and pyramidal feature hierarchy, which leads to better performance in small-scale object recognition. In contrast, networks with encoder–decoder structures can accurately represent image contour features but have poor representation abilities for small-sized ground objects. This happens because the encoder–decoder networks use shallow and fine feature maps of the backbone to obtain small-scale semantic information, which naturally leads to the poor ability of obtaining small-scale semantic information and decreased segmentation accuracy. To leverage the benefits of the pyramidal and encoder–decoder networks and avoid their respective drawbacks, we propose a novel encoder–decoder network, which will be referred to as EDPNet, where the pyramidal module is embedded into the network to jointly encode the augmented features with rich semantics by multi-scale representations and aggregate discriminative features for accurate image contour delineation.

### 2.5. Novel Contributions

In the context of the proposed EDPNet operational framework, the primary novel contributions of our paper can be summarized as follows:

(a) We propose EDPNet, a novel convolutional neural network tailored for the semantic image segmentation task. EDPNet is a hybrid network that combines the encoder–decoder structure and the pyramidal representation at multiple scales to aggregate context-aware augmented features for precise boundary delineation.
(b) We introduce an auxiliary loss function at the end of the proposed network’s feature extraction module to supervise the model output. Auxiliary loss can optimize the parameters of the convolution layers which are far away from the main loss and accelerate learning progress for the main task, significantly improving the network’s overall performance.

This paper is organized as follows. After the first two introductory sections, Section 3 describes the entire architecture of the proposed EDPNet, including the encoding module, pyramidal feature aggregation module, and decoding module. In Section 4, we present and analyze the experimental dataset and performance evaluation results of semantic image segmentation from four benchmark image datasets. Finally, Section 5 concludes the paper along with a few suggestions for future research topics.

## 3. Methodology

### 3.1. EDPNet Framework

**Figure 1:**
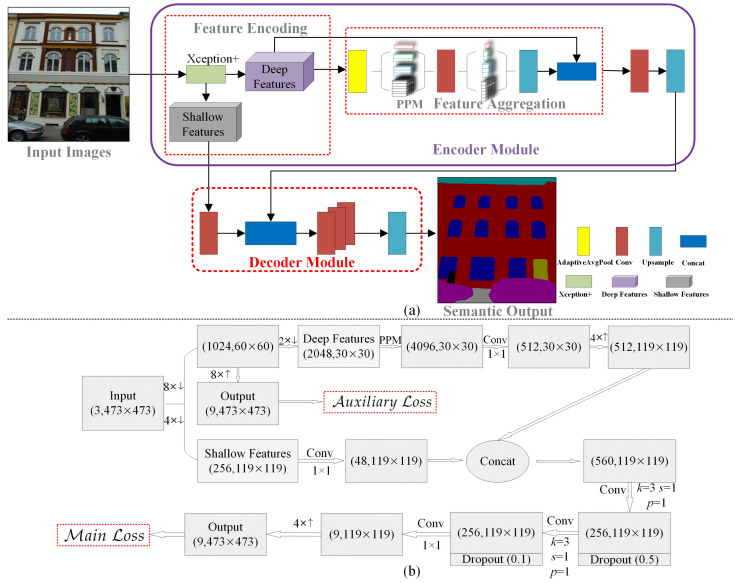
Framework of the proposed EDPNet. Subfigure (**a**) represents the basic components of the proposed network, and (**b**) represents the corresponding intermediate feature maps. Note that the symbols “↑” and “↓” denote upsampling and downsampling operations.

The proposed hybrid deep learning framework EDPNet combines encoder–decoder architecture and pyramidal representation for semantic image segmentation. As shown in Figure 1, the input images are encoded by an enhanced Xception network (see Section 3.2) to obtain 16× deep and 4× shallow feature map representations. After that, the 16× deep feature maps are summarized by an adaptive average pooling and then delivered into a pyramidal pooling module, which generates context-aware and aggregated features by representing the deep feature maps at four different pyramidal scales. The outputs of the encoder are then fed into the decoder branch for image restoration. During decoding, the encoder’s deep features are fused with 4× shallow features derived by an enhanced Xception so that the low-level details can be retained as much as possible. The fused feature maps are then decoded by three consecutive convolutions and one upsampling. The detailed pipeline’s modules and their corresponding feature map variations are shown in Figure 1a,b.

Currently, most semantic image segmentation networks utilize softmax activation along with cross-entropy loss function in Equation (1) to adjust model weights iteratively with the aim of minimizing the cross-entropy loss

(1) $$\begin{array}{c} Loss\left(y,\tilde{y}\right)=-\frac{1}{N}\sum_{x}\left[y\log\tilde{y}+\left(1-y\right)\log\left(1-\tilde{y}\right)\right] \end{array}$$

where, *y* represents the ground-truth label, and $\tilde{y}$ represents the probability values of the predictions. $Loss(y,\tilde{y})$ measures the discrepancy between the ground truth and the predictions. *N* denotes the number of samples, and *x* denotes the number of semantic classes. It should be noted that the proposed EDPNet comprises of numerous convolutional layers, and thus requires tuning a large number of weights of neural network layers during the process of backpropagation. Therefore, to optimize the training process, especially for fine-tuning weights of convolutional layers that are far away from the loss function, more than one loss function is necessary. To prevent slow training because of the vanishing gradient issue and promote learning of each module layer, and motivated from GooLeNet [54] and PSPNet [41], we introduce an auxiliary loss after the feature encoding block to help the network to reduce the vanishing gradient for earlier layers and stabilize the training without influencing learning guided by the main loss function. The proposed loss function, which combines main loss and auxiliary loss, is given below:

(2) $$\begin{array}{c} Loss\left(y,{\tilde{y}}_{main},{\tilde{y}}_{aux}\right)=Main\_Loss\left(y,{\tilde{y}}_{main}\right)+\mu *Aux\_Loss\left(y,{\tilde{y}}_{aux}\right)\\ s.t.\;Main\_Loss=Loss(y,{\tilde{y}}_{main})\\ Aux\_Loss=Loss(y,{\tilde{y}}_{aux}) \end{array}$$

where, ${\tilde{y}}_{main}$ and ${\tilde{y}}_{aux}$ represent the predicted results by main and auxiliary branches. *y* represents the ground truth. Parameter μ denotes the weight of the auxiliary loss, which is used to balance the main loss and auxiliary loss during the learning process by backward propagation. In our case, the value of μ is set between 0 and 0.9. The main cross-entropy loss in our network is calculated by comparing the ground truth labels with the predicted feature maps generated by the main branch of the EDPNet, while auxiliary cross-entropy loss is calculated by comparing the ground truth with the predicted feature maps that have undergone 8× downsampling by the Xception+ and subsequent upsampling by the same times using bilinear interpolation. Experimental results demonstrate that the auxiliary loss helps optimize the learning process and yields remarkable performance in semantic image segmentation.

### 3.2. Encoding Module

The encoding module aims to extract features and achieves aggregated feature representations at multiple scales. The proposed encoder contains two parts, namely the enhanced light-weighted Xception+ network and the pyramid pooling module. Xception+ extracts basic semantic features of the inputs, while the pyramid pooling module uses these features to obtain multiple feature representations that are more augmented and discriminative for semantic image segmentation.

**Figure 2:**
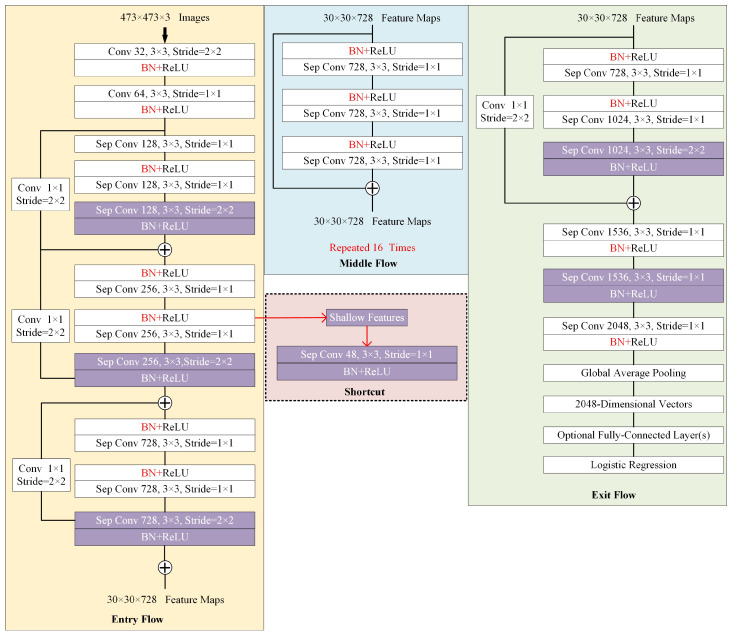
Feature map extraction by an enhanced Xception network called Xception+. Note that our enhanced blocks are highlighted by the purple backgrounds. We add the shortcut block, which aims to retain shallow feature maps and decrease the number of feature channels.

(1) Feature Extraction: As the basic structure of the semantic segmentation network, the feature extraction module directly determines deep-level semantics and low-level appearance information embedded in the extracted feature maps. Most semantic image segmentation networks employ classic networks such as VGG [55], ResNet [44], and Xception [56] as backbones for feature encoding and integration. These networks have demonstrated excellent performance in various recognition problems based on the large-scale ImageNet benchmark [57] and prove the abilities of feature representation and aggregation. In addition, these networks have a powerful transfer learning ability by loading a pre-trained ImageNet model to accelerate convergence and improve network performance. As light-weighted Xception is more efficient and exhibits powerful encoding ability compared with VGG and ResNet, in the proposed EDPNet, we adopt Xception as a backbone for initial feature extraction and representation. Inspired by works [56,58], we enhance the original Xception module to further strengthen the feature encoding ability. The enhanced module, called Xception+, includes the following significant enhancements: (*i*) The original Xception network uses downsampling operators such as stride convolutions or pooling layers to encode the feature representations. While these enlarge the receptive field size to perceive high-level semantics, they also increase the loss of spatial information, especially for deep feature maps. To guarantee a large-sized receptive field and relieve the spatial information loss, we substitute the Xception’s pooling structures with depthwise separable convolutions with 3 × 3 filters and a stride of 2 to retain more details. The changes are illustrated by rectangles with purple backgrounds in Figure 2. (*ii*) We draw out a shortcut block from “Entry Flow” block in Figure 2 to retain shallow features with detailed appearance information and make fusions with encoder outputs (see Figure 1) with rich semantics.
(2) Feature Aggregation: After extracting feature maps using Xception+, we further enhance global feature representation at different contextual scales by embedding a pyramidal aggregation module into EDPNet. Pyramidal structures can better encode global context feature maps to relieve semantic image segmentation problems, such as mismatched relationships, confusion categories, and inconspicuous classes. In addition, by the multiple-level representations, the aggregated features can significantly parse complex scenarios, especially for the scenery context with different shape sizes of geographical entities because the multi-level representation is easier to retain global contextual information compared to tandem pooling networks.

**Figure 3:**
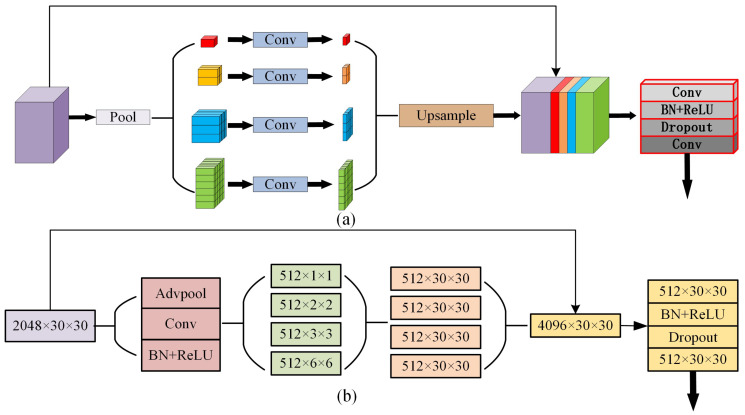
Pyramidal pooling module (PPM) proposed by Zhao et al. [41]. Subfigure (**a**) represents PPM structural modules, and (**b**) corresponds to the feature maps of the PPM.

In the proposed EDPNet, we directly integrate the PPM module [41] into our encoder branch for simplicity to achieve multiple-level representations. We use four levels of features maps with 1 × 1, 2 × 2, 3 × 3 and 6 × 6 pools to encoder scenery contextual information, followed by 1 × 1 convolutions to reduce the channel to 1/4 of the original one. After that, we upsample low-dimensional feature maps into the same size as the input feature maps using transpose convolution. Different levels of features are concatenated with the original features, and 1 × 1 convolution is subsequently used to squeeze the original channels to produce the final pyramidal global feature maps. The detailed structures of the PPM module are illustrated in Figure 3.

### 3.3. Decoding Module

The decoder module is responsible for restoring the coarse resolution feature maps to the exact size of fine-grained input resolutions by progressively adopting upsampling strategies. The most commonly used upsampling techniques are transposed convolution, nearest neighbor interpolation, bilinear interpolation, and bicubic interpolation. Although these methods restore some of the spatial information of the input during the task of semantic image segmentation, it is important to note that upsampling cannot completely restore the information loss in the encoder due to deep stacking convolutional and pooling layers. This implies that the fine-grained contours of the geographical objects in the images cannot be completely depicted, thereby weakening the accuracy of the semantic image segmentation. To solve this problem to some extent, skip connection is the most effective way to compensate for information loss by fusing shallow, fine, appearance information with deep, coarse, and semantic-rich feature maps to augment the details of feature maps. In the proposed EDPNet, we use a simplified version of the mechanism of skip connection to guarantee the accuracy and efficiency of decoding. We construct two types of feature maps: deep and shallow feature maps by the Xception+ module. The deep features are further represented by the pyramidal encoding to obtain aggregated and more augmented feature maps, which are then restored in the same size as the original input with the help of shallow feature fusions.

**Figure 4:**
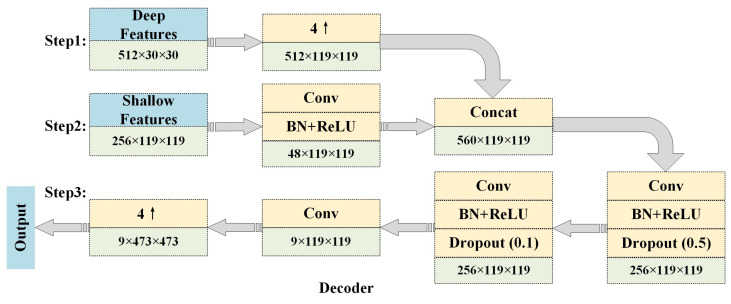
Decoder structure in EDPNet. Note that rectangles denoted by light blue colors are input and output. Rectangles with yellow colors represent the specific operations, while light green rectangles denote the corresponding feature maps.

In our EDPNet, we use the proposed encoder to shrink the input images into 1/16 feature maps of the original size with 512 channels. 4× bilinear interpolation is followed to obtain 1/4 dimension of the input to finalize the encoding process. The encoded feature maps are then put into the decoding step where shallow feature maps of the same dimensions are concatenated with the encoded features to form aggregated feature maps of dimension 560 × 119 × 119. The aggregated feature maps are deeply fused through three successive convolutions and one pool operation to learn feature maps with rich semantics and spatial contextual details. The detailed decoding process is shown in Figure 4.

## 4. Performance Evaluation

### 4.1. Dataset Specification

**Figure 5:**
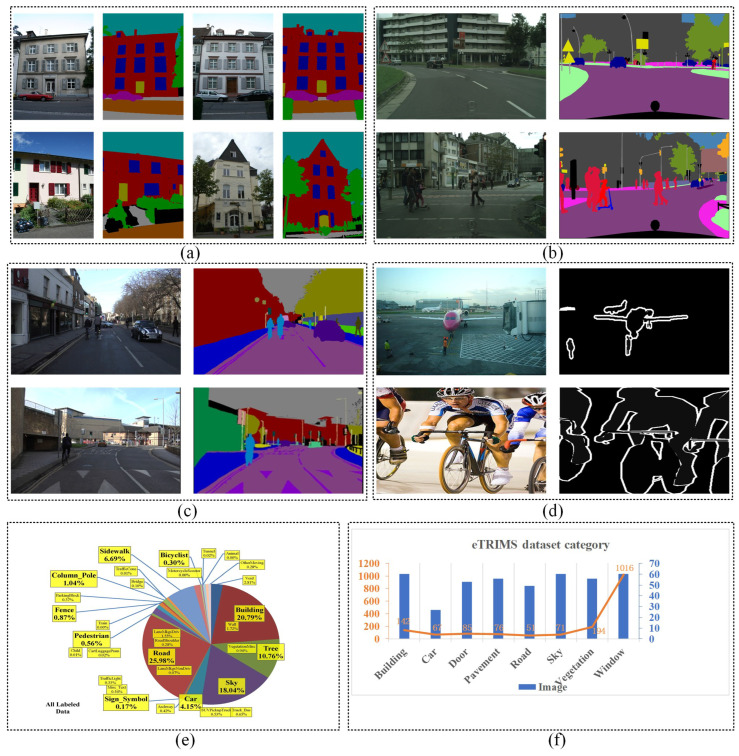
The open source benchmarks used in our paper for experimental evaluations. Subfigures (**a**–**d**) are part of benchmarks randomly selected from eTRIMS [59], Cityscapes [60], CamVid [61] and PASCAL VOC 2012 [62], respectively. Subfigure (**e**) [61] and (**f**) are the distributions of different object categories in CamVid and eTRIMS datasets, respectively. Each bar in (**f**) displays the number of images belonging to each category, while the curved orange line represents the object numbers within each class in the eTRIMS dataset.

To evaluate the effectiveness of the proposed EDPNet, we utilize datasets of eTRIMS [59], Cityscapes [60], CamVid [61], and PASCAL VOC 2012 [62] as the experimental datasets. As shown in Figure 5, the eTRIMS dataset is a building façade dataset with 8 object classes and includes 60 annotated images. Among them, we have selected 44 images as the training set, 6 images as the validation set, and 10 images as the testing set to conduct semantic segmentation of structural building components. The eTRIMS benchmark is an ideal dataset for interpreting images of man-made building façade scenes as buildings are important components and the most significant man-made geographical entities for creating digital twins for cities. Cityscapes dataset focuses on semantic parsing of urban street scenes, which includes 5000 images with 19 object categories. In our experiment, 2975 images were randomly selected as training data and 500 images were used as validation data. Another dataset is the CamVid dataset, which is a fine-grained annotation for road/driving scene understanding dataset of video images for road scenes. This dataset is the first collection of videos with object class semantic labels. For the CamVid dataset, the most common 12 categories of objects are chosen from this dataset to evaluate the segmentation accuracy. We selected 567 images as the training set, 63 images as the validation set, and 71 images as the testing set. The final dataset used in this paper is the PASCAL VOC 2012 dataset which was set up to recognize objects in realistic scenarios from a variety of visual object types and has been widely used in image classification, semantic segmentation, and object detection. The PASCAL VOC 2012 dataset has 21 object categories including background semantics and consists of 10582 images in the training set and 1449 images in the validation set.

### 4.2. Experimental Environment

The proposed EDPNet was trained and predicted based on a deep learning server using an NVIDIA GTX 3090 24 GB GPU (NVIDIA Corporation, Santa Clara, CA, USA) and a 3.00 GHz Intel Core i9-10980 XE CPU (Intel Corporation, Santa Clara, CA, USA), 64 GB main memory. We implement the programming based on the Pytorch-gpu 1.7.1 framework in Python 3.6 language. The NVIDIA GTX 3090 GPU is powered by CUDA 11.1. SGD optimizer is used to update the model’s parameters.

### 4.3. Evaluation Metrics

Pixel accuracy (PA), mean pixel accuracy (mPA) and mean intersection over union (mIoU) are used as the metrics for the experimental evaluations. The PA metric is calculated by dividing the correctly segmented pixels by the total pixels in the ground truth image, while mPA represents the class average accuracy. These metrics have computed using the following formulas:

(3) $$\begin{array}{c} PA=\frac{\sum_{i=0}^{k}p_{ii}}{\sum_{i=0}^{k}\sum_{j=0}^{k}p_{ij}} \end{array}$$

(4) $$\begin{array}{c} mPA=\frac{1}{k+1}\sum_{i=0}^{k}\frac{p_{ii}}{\sum_{j=0}^{k}p_{ij}} \end{array}$$

where, *k* is the number of semantic classes; $p_{ij}$ represents the number of pixels that are incorrectly predicted as class *j* but are labeled as class *i* in the ground truth. In contrast, $p_{ji}$ represents the number of pixels that are mistakenly classified as class *i* but are labeled as class *j*. $p_{ii}$ corresponds to the total number of true positive pixels for class *i*.

Intersection over union (IoU) represents the ratio of intersected pixels to the union of the pixels between the prediction and ground-truth images, which can assess the model’s semantic segmentation capability for a specific class in an image. Assuming that there are *k* classes in the dataset to be segmented, the IoU of the semantic class *i* is defined as follows:

(5) $$\begin{array}{c} {\left(IoU\right)}_{i}=\frac{p_{ii}}{\sum_{j=0}^{k}p_{ij}+\sum_{j=0}^{k}p_{ji}-p_{ii}} \end{array}$$

where, $p_{ij}$ denotes the pixels belonging to class *i* but classified into class *j*, and $p_{ii}$ indicates the number of correctly classified pixels. Furthermore, based on each class’s IoU, the mean intersection over union (mIoU) is adopted to evaluate the multi-class semantic segmentation accuracy, which can be calculated as follows:

(6) $$\begin{array}{c} mIoU=\frac{1}{k+1}\sum_{i=0}^{k}{\left(\mathrm{IoU}\right)}_{i} \end{array}$$

### 4.4. Parameters Setting

The parameters of the EDPNet were obtained using the eTRIMS dataset, and then the determined parameters were applied to the semantic segmentation of the other three datasets. The input images are cropped to a size of 473 × 473 to better adapt to pyramidal representations in the PPM module. To avoid overfitting, a comprehensive data enhancement method was applied to the preprocess of all datasets, including resizing, mirroring, adding rotation between −10° and 10°, and adding Gaussian blur to the datasets in a random manner. The parameters of the EDPNet and the values used in our experiments can be found in Table 1.

**Figure 6:**
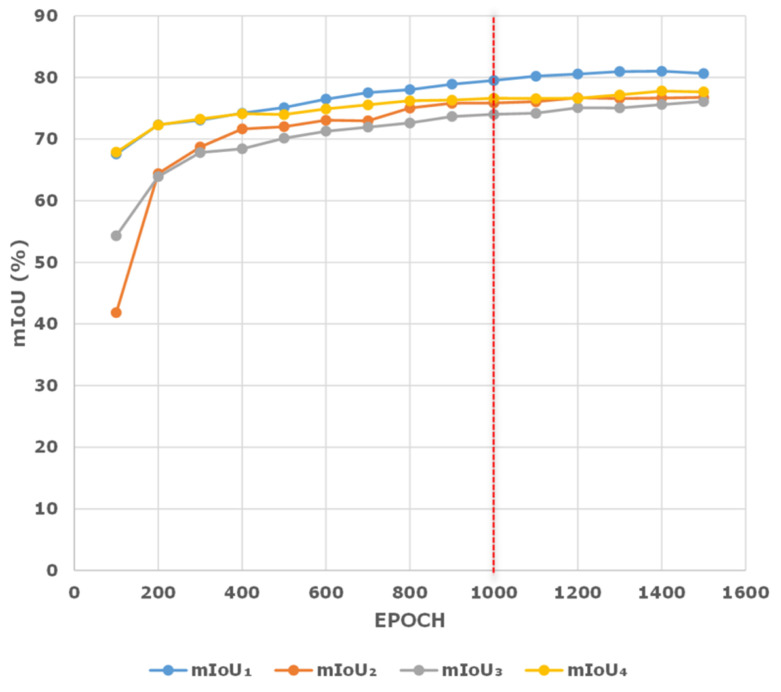
Network performance trained at different epochs. mIoU_1–4_ represents the semantic segmentation accuracy of the eTRIMS verification set carried out 4 times.

In Table 1, two parameters, namely loss weight factor μ and the training epoch Epoch are sensitive to extreme values. In our paper, we achieve statistically accurate results by repeatedly conducting experiments using different values of Epoch and μ to obtain the optimized values in the eTRIMS dataset. As shown in Figure 6, it can be observed that the mIoU of the validation set basically gives better results at around 1000 epochs. If we continually increase the epoch values, the polylines become saturated with a narrow margin of mIoU improvement, which implies that the training epochs at around 1000 is indeed a good balance between predicted performance and training efficiency.

**Figure 7:**
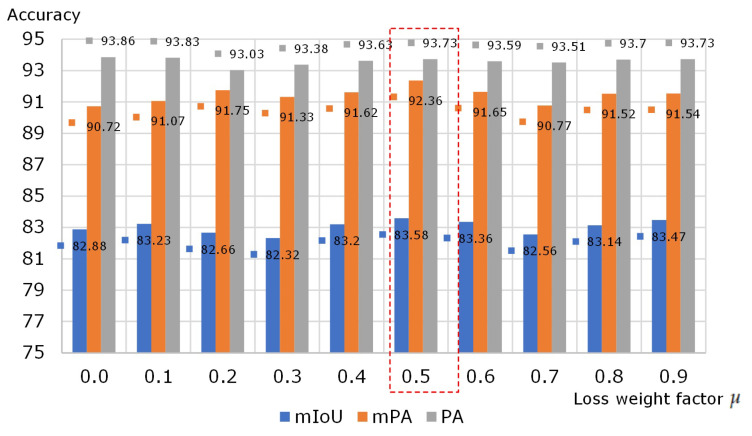
Performance evaluation results for the eTRIMS validation set using different values of loss weight factor μ.

To further improve the segmentation accuracy of the EDPNet, the auxiliary loss was added to enhance the training efficiency. The effects of different values of loss weight factor μ on the segmentation results are displayed in Figure 7, where μ takes values in the range from 0.0 to 0.9. It can be observed that extremely large or small values of μ may decrease the accuracy of the network. By trial-and-error experiments, we found that the value of 0.5 can best balance auxiliary loss and main loss as the highest accuracy of 83.58% mIoU can be achieved. Note that the results in Figure 7 were obtained when the eTRIMS dataset was trained at 1000 epochs and pre-loaded with weights trained on ImageNet. All subsequent experiments were conducted with μ = 0.5 as a fixed parameter.

### 4.5. Ablation Experiments

Ablation experiments have been conducted based on the eTRIMS dataset to further evaluate the effects of two feature extraction modules, three pooling strategies, a multi-scale feature pyramidal representation module, and a decoder module embedded into the proposed EDPNet. The quantitative results of the ablation study are shown in Table 2, where the baseline is defined as ResNet50-based FCN; ‘P1’ and ‘P1236’ represent feature maps of sizes $\left\{1\times 1\right\}$ and multiple representations by $\left\{1\times 1,2\times 2,3\times 3,6\times 6\right\}$ in the PPM module. ‘MAX’, ‘AVE’, and ‘ADAVE’ represent the maximum, average, and adaptive average pooling for obtaining multi-scale feature representations in the PPM. ‘DC’ denotes the decoder module and ‘Size’ indicates weight size.

In Table 2, we use two encoding networks, i.e., ResNet50 and Xception+ to encode the initial feature maps of the input images. ResNet50 solves the model degradation problem well by continuously stacking residual learning modules. While Xception+ uses depthwise separable convolution combined with a skip connection mechanism to speed up the convergence process significantly. Although these two models have a powerful encoding ability, compared with EDPNet-c2, the training time of EDPNet-c4 is reduced by 33%.

Next we will highlight the capabilities of pyramidal representations and the decoder. In our case, EDPNet-c6∼EDPNet-c8 are appropriately configured with multi-scale PPM modules in order to obtain higher mIoUs than EDPNet-c3∼EDPNet-c5. This happens due to the fact that the PPM module adopts different scale pooling operations on the input map to obtain four feature maps with different sizes. As such, it aggregates the contextual features, thus effectively increasing the segmentation accuracy. Besides, the addition of the ‘DC’ module can increase the network model training accuracy, as can be verified by the mIoUs of EDPNet-c9∼EDPNetc-11 in Table 2. This improvement is attributed to the integration of 4-fold down-sampled feature maps in the ‘DC’ module, which greatly preserves the spatial features of the image and facilitates the dedicated segmentation.

In the pyramidal feature representation, we use three pool strategies, namely average pooling layer, maximum pooling layer, and adaptive average pooling layer to finalize multi-scale feature representations. As can be seen from Table 2, the pooling type affects the segmentation accuracy, i.e., the mIoU accuracy increases from 79.1% for the ‘EDPNet-c11’ to 79.7% for the ‘EDPNet-c10’ and then to 80.2% for the ‘EDPNet-c9’. This improvement demonstrates that the adaptive averaging pooling captures the semantic information of regions more effectively.

We also quantitatively evaluate the semantic segmentation accuracy of the eTRIMS’s individual class through an ablation study. As shown in Table 2, ‘EDPNet-c2’ shows the best results on three categories, i.e., ‘Pavement’, ‘Road’ and ‘Sky’; however, its IoUs of the classes for ‘Car’ and ‘Door’ categories are relatively poor. This is because class ‘Car’ has few training samples and the object size of class ‘Door’ is very small in the image, resulting in insufficient training of ‘EDPNet-c2’. After adding the ‘P1236’ module, the segmentation accuracy of ‘EDPNet-c2’ for ‘Car’ is increased because the depthwise separable convolution and pyramid structure can retain more details. With the addition of the ‘DC’ module, the segmentation accuracy of ‘Door’ and ‘Windows’ has significantly improved. This indicates that the‘DC’ module has better recognition and segmentation ability on small-sized objects. Since ‘EDPNet-c9’ achieves good mIoU performance and takes all categories into account, ‘EDPNet-c9’ has been selected as the final architecture of the EDPNet network proposed in this paper. For simplicity of the presentation, we will continue to use the EDPNet instead of EDPNet-c9 for the remainder of this paper.

To further verify the effectiveness of ‘Xception+’ and ‘DC’ modules, we have compared EDPNet, EDPNet-c6, and PSPNet on the eTRIMS validation images. For this we have chosen the PSPNet because EDPNet and PSPNet use the same pyramidal feature representation modules. Compared with PSPNet, EDPNet-c6 has a similar network architecture except for the backbone of the feature encoding module. EDPNet-c6 uses Xception+ as the feature encoding backbone, while PSPNet adopts ResNet50 as a backbone. To make fair comparisons, EDPNet-c6 and EDPNet also use 8-fold downsampling so that they are consistent with 8-fold downsampling in PSPNet. The semantic accuracy of these three network models on the eTRIMS validation images is shown in Table 3. Compared with the PSPNet model, the overall mIoU accuracy of EDPNet-c6 and EDPNet have improved by 1.7% and 2.4%. In addition, for most classes, EDPNet-c6 and EDPNet achieve the highest accuracy. This proves that Xception+ indeed outperforms ResNet50 model, which is adopted in PSPNet as a backbone. However, EDPNet-c6 has the lowest 66.4% IoU for ‘Pavement’ segmentation due to relatively few training samples and complex features of this class. Despite this, EDPNet still obtains the highest 73.5% IoU pavement segmentation, which implies that EDPNet with the addition of the ‘DC’ module has better spatial segmentation capabilities and can strike a better performance with a smaller training dataset.

### 4.6. Prediction Results of Different Networks on the eTRIMS Dataset

Seven mainstream semantic image segmentation models are compared on the eTRIMS dataset. Two evaluation metrics, i.e., mIoU and training time are used for quantitative measurements, and the results are summarized in Table 4. It can be observed that the proposed EDPNet achieves the highest 83.6% mIoU and takes 125 minutes of total training time. Compared with the PSPNet, which ranks in second place with 83.0% mIoU, the training time of the proposed EDPNet is reduced by 28.2%. Compared with the most lightweight and efficient semantic segmentation network LR-ASPP [64], EDPNet’s mIoU is improved by 24.3%.

**Figure 8:**
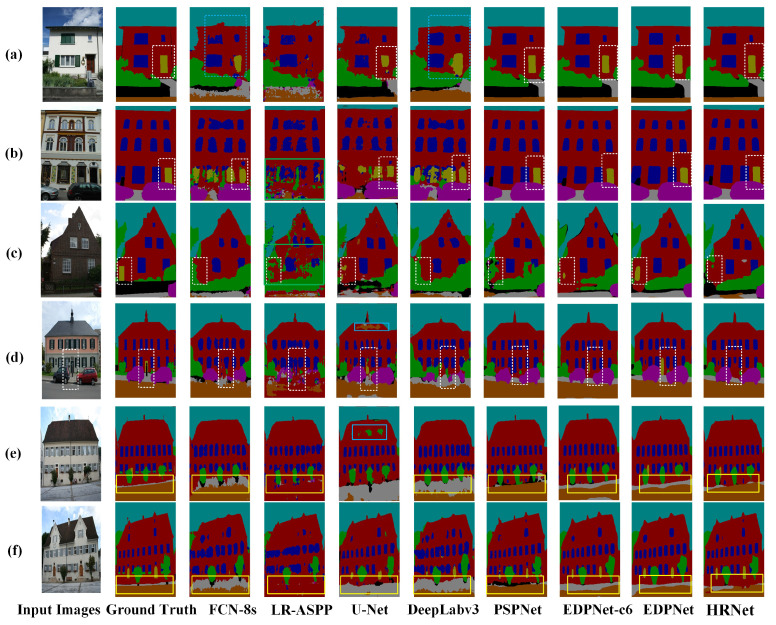
Semantic segmentation results obtained by seven different networks on the eTRIMS test set. The represented buildings depicted in subfigures (**a**–**f**) were selected from the eTRIMS dataset.

The qualitative semantic segmentation results of EDPNet and the other seven models on the eTRIMS dataset are presented in Figure 8. The segmentation results of the FCN-8s and DeepLabv3 miss a lot of component details in the building façade, as marked in the dashed blue rectangles in Figure 8a, thereby causing the door and window edges to be blurred and incomplete. There are many segmentation errors generated by the LR-ASPP model as well, such as the wrong segmentation of doors, windows, and vehicles, as demonstrated in the green rectangles in Figure 8b,c. For the U-Net, a few commission errors occur at the building rooftops, as shown by the solid blue boxes in Figure 8d,e. It is also noted that the HRNet, it is not able to recognize categories with fewer labels in the training set, such as the “Door” in Figure 8c,d. On the contrary, in general, EDPNet achieves excellent semantic segmentation on regular shapes and fully trained classes, such as “Door” and “Window”, as is denoted by white dotted rectangles in Figure 8a–d. It also shows accurate segmented results on irregular and inadequately trained categories, such as “Pavement” and “Vegetation”, denoted by the yellow rectangles in Figure 8e,f. The segmentation comparisons show the advantages of EDPNet for maintaining spatial details of the building façade’s components in the eTRIMS dataset.

### 4.7. Evaluation of EDPNet on the Cityscapes Dataset

We have also compared the proposed EDPNet with seven other networks, including FCN-8s, LR-ASPP, PSPNet, U-Net, DeepLabv3, HRNet and EDPNet-c6 on the Cityscape dataset. Considering a large volume of training images, the mIoU and training time of all seven models at 500 training epochs have been calculated. The input images are cropped to 713 × 713 for PSPNet and 761 × 761 for others. The quantitative results are shown in Table 5. It can be seen that PSPNet has achieved the highest mIoU of 76.0%. Although the mIoU of EDPNet-c6 and EDPNet is 0.4% and 4.8% lower than that of PSPNet, their training time is 47.5% and 41.2% less than PSPNet. In addition, for the dominant classes such as ‘Road’, ‘Vegetation’, ‘Sky’ and ‘Car’, which are adequately trained, these eight models have no significant differences in accuracy for these dominant classes. However, for the scene objects with small scale, irregular and/or complex shapes, e.g., ‘Wall’, ‘Fence’, ‘Motorcycle’ and ‘Rider’, they tend to have inadequate training and the segmentation accuracy for these classes is extremely low, especially for the U-Net and DeepLabv3 networks.

**Figure 9:**
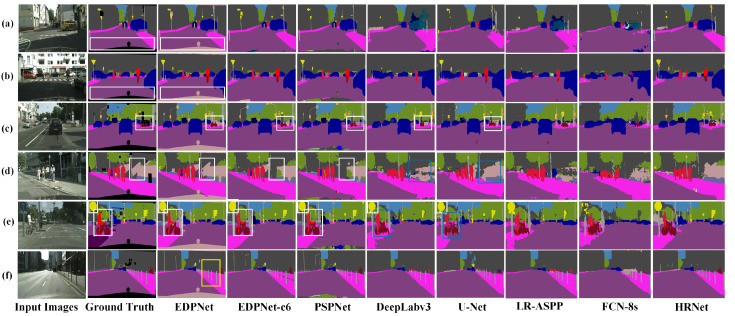
Semantic segmentation results predicted by different networks on the Cityscapes validation set. The scenes depicted in subfigures (**a**–**f**) were randomly selected from the Cityscapes validation dataset.

The qualitative prediction results are shown in Figure 9. Among these eight models, the segmentation results obtained by DeepLabv3 and U-Net are coarse at the edges, which is demonstrated in the blue rectangles in the last two columns in Figure 9. The segmentation results of PSPNet, EDPNet-c6, and EDPNet are more accurate overall, but the EDPNet model outperforms others in identifying the window positions, as depicted in the white rectangles in Figure 9a,b. This is because the 4-fold downsampling of the ‘DC’ module in the EDPNet retains more spatial information, which in turn supplements the semantic details as well as feature information lost during iterative learning of the network. In addition, the segmentation results of the EDPNet have more sharp and clear edges and less over- and/or under-segmentation compared to the PSPNet model, as is evident in the white rectangles in Figure 9c–e. Besides, from the roadside railing in the yellow rectangles in Figure 9f, it can be seen that the EDPNet obtains more complete and sharp results for these small-sized objects. In short, through the visualization results on the Cityscapes validation set, EDPNet shows its advantages in acquiring semantic information and maintaining spatial details of urban street scenes.

### 4.8. Evaluation of EDPNet on the PASCAL VOC 2012 Dataset

The images of the PASCAL VOC 2012 dataset are cropped to 473 × 473 as inputs, and the training epoch is set to 500 to balance training efficiency and predicted accuracy. The quantitative comparisons are shown in Table 6. It can be seen that EDPNet has the highest 73.8% mIoU, and achieves the highest IoU in most categories. Compared with PSPNet, EDPNet has a 4.3% increase in mIoU and a 45.6% decrease in training time. Furthermore, FCN-8s, LR-ASPP, DeepLabv3 and U-Net perform poorly, whose mIoUs are under 60%, and especially fail in segmenting complex and irregular objects. Although HRNet and PSPNet achieve higher mIoUs of 73.4% and 69.5%, compared to other models, they require significantly longer training time. This clearly verifies that the EDPNet can better balance the accuracy and training efficiency compared with other network models.

**Figure 10:**
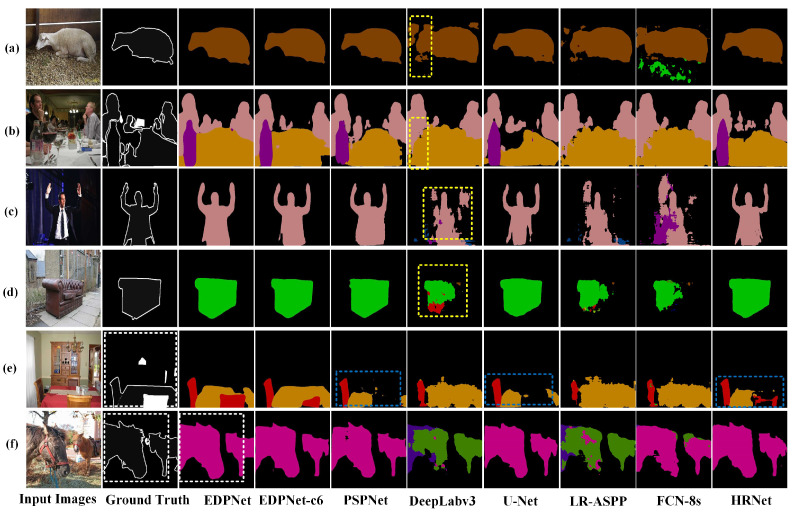
Semantic segmentation results obtained for different networks on PASCAL VOC 2012 validation dataset. The images depicted in subfigures (**a**–**f**) were randomly selected from the PASCAL VOC 2012 validation dataset.

Next, the segmentation results are presented in Figure 10. From a qualitative perspective, we find that DeepLabv3 suffers from misclassification and ambiguity, as shown in the yellow rectangles in Figure 10a–d. This is due to the fact that DeepLabv3 restores the original resolution by 8×, upsampling the feature map directly, which loses much spatial information. The segmentation results of UNet, PSPNet and HRNet have the problem of under-segmentation and objects with occlusions are often missed, as depicted in the blue dashed rectangles of Figure 10e.

By contrast, EDPNet and EDPNet-c6 have more clear and sharp segmentation results, such as the “Bottle” in Figure 10b and the “Diningtable” in Figure 10e. This is probably due to the stronger feature encoder capability and feature map restoration by incorporating shallow features with fine-grained appearance information. Compared with the EDPNet-c6, EDPNet’s decoder module, with bilinear interpolation upsampling and the supplement of shallow features, brings more bright edge details, as seen in the “horse” segmentation in Figure 10f.

### 4.9. Evaluation of EDPNet on the CamVid Dataset

To further verify the capability of the proposed EDPNet for parsing complex and fine-grained road scenes, we have selected 12 common categories in the CamVid dataset and have compared the accuracy and training efficiency of EDPNet with seven other methods. For the fairness of the experiment, all the images fed to the five networks are resized to 761 × 473, and the training epoch is set to 1000.

The quantitative semantic segmentation results are shown in Table 7. For the dominant objects, such as ‘Building’, ‘Road’, ‘Sidewalk’, ‘Sky’ and ‘Tree’, the IoUs of the five networks are very close. However, EDPNet and PSPNet obtain high segmentation accuracy in small-sized categories with insufficient training data and complex spatial features, such as ‘Car’, ‘Fence’, ‘Pedestrian’ and ‘Wall’. Although the accuracy of EDPNet is 1.8% lower than that of PSPNet, the training time is reduced by 44.7%, again showing a good balance between accuracy and training efficiency on the CamVid test dataset.

**Figure 11:**
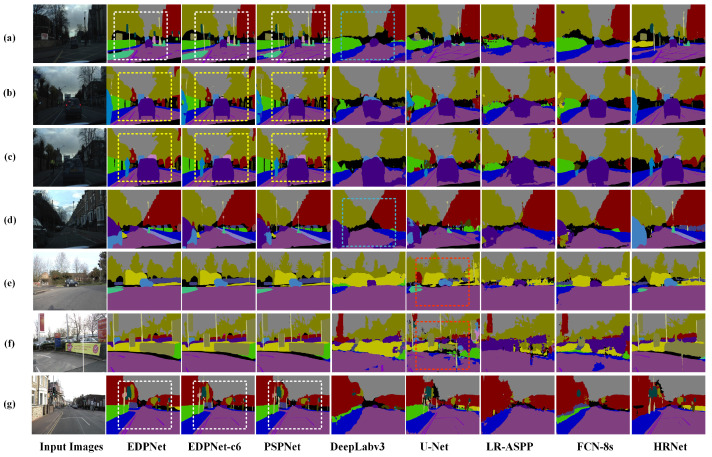
Semantic segmentation results obtained by different networks on the CamVid test dataset. The scenes depicted in subfigures (**a**–**g**) with varying complexities were selected from the CamVid test dataset.

The validation images and corresponding segmentation results are shown in Figure 11, where various road scenes under different weather conditions are selected. As displayed in Figure 11a, the scene is captured at a road junction under weak illumination. The whole scene contains many small and medium size objects, and the distant vehicles and utility poles pose great challenges for semantic image segmentation. Figure 11b–d are also captured in an environment with poor illumination. The biggest challenge for these images is to correctly segment distant cars located at different locations on road surfaces. In Figure 11e–g, these scenes have high illumination and diverse objects; however, the distant signal lights, street light poles, cars, and trees increase challenges for road scene segmentation.

After qualitative comparisons of five segmentation models, we find that DeepLabv3 can only effectively segment small objects with adequate training data. For example, the street lamps in blue dashed rectangles in Figure 11a,b have omission errors. For the U-Net model, there exist serious commission errors in larger object segmentation. Such problems can be observed in Figure 11e,f, where many pixels of roadside trees and billboards in the red dashed rectangles are mistakenly segmented into other categories. In the case of HRNet, certain inaccurate segmentation results may occur, as evidenced by the vehicle segmentation error in Figure 11d and the wall identification error in Figure 11g. After comparing the segmentation results of PSPNet, EDPNet-c6, and EDPNet under different kind of scenes, it can be concluded that EDPNet achieves the best overall segmentation results. As displayed in the white dashed rectangles of Figure 11a,g, the segmentation results of EDPNet have the sharpest edges on the whole scene, even if there exist many scattered objects. In addition, EDPNet has the complete segmentation of vehicles even under weak illumination weather conditions, as indicated in the yellow dashed rectangles in Figure 11b,c. This is because EDPNet uses the ‘Xception+’ feature extraction module, where depthwise separable convolution can effectively preserve more details. In addition, the ‘DC’ module can also enhance the spatial information extraction ability of the network and thus refine the expression of object edges.

## 5. Conclusions and Suggestion for Further Research

In this paper, we have proposed a novel network EDPNet, which combines the encoder–decoder and the pyramid structure for accurate semantic image segmentation. Using four datasets, it has been demonstrated that the performance of the proposed EDPNet is at par with or even better than other state-of-the-art classical CNN architectures. Various performance evaluation results have shown that, as compared to the SOTAs, the EDNet has the highest computational efficiency and at the same time makes a good balance between semantic accuracy and training efficiency. In addition, from a qualitative perspective, EDPNet can parse complex urban road scenes with diverse and extremely small-sized shape features (such as pedestrians, cars, light poles, billboards, and roadside railings) with sharper target boundaries.

Although the proposed EDPNet is lightweight, highly efficient, and accurate, there are some drawbacks that should be considered. The pyramid module used in this article is an improvement on the PPM module in PSPNet, so the performance of the multiple-resolution representation is not exempted from the limitations of the original PPM. Although our network is lightweight and robust for most of the open-source benchmarks, one should be aware that our network is still slightly inferior to the latest but complicated and cumbersome semantic segmentation models, such as TAGNet [65] and CTNet [66]. In our future work, we will consider designing a more advanced pyramid structure for advanced multi-scale representation and adding some more appropriate mechanisms to make a tradeoff between the model’s accuracy, efficiency, and compaction. We also plan to expand our network to other 2D recognition tasks, including object detection and panoptic segmentation, which can leverage the potential of the proposed EDPNet.

## Figures and Tables

**Table 1:** Training parameters of EDPNet for eTRIMS datasets.

Parameter	Recommended Value/Configuration
Learning rate (lr)	0.001
Loss weight factor (μ)	0∼0.9
Momentum	0.9
Weight decay	0.0001
Batchsize	4
Loss	Cross-entropy Loss
Epoch	1000
lr_scheduler	Poly [63]

**Table 2:** Ablation performance of the eTRIMS validation dataset.

Models	Configurations	mIoU (%)	Time (min)	Size (MB)	Building	Car	Door	Pavement	Road	Sky	Vegetation	Window
EDPNet-c1	ResNet50+FCN Baseline	54.7	120.0	282.9	81.2	56.0	29.4	26.0	67.9	64.9	61.6	55.4
EDPNet-c2	ResNet50+P1 +MAX	80.5	169.0	393.0	89.9	87.8	72.6	71.6	93.9	97.3	72.0	79.4
EDPNet-c3	‘Xception+’+ P1+ADAVP	76.3	110.0	507.7	88.3	91.2	70.5	54.7	82.6	96.8	72.0	71.7
EDPNet-c4	‘Xception+’+P1 +MAX	77.2	113.0	507.7	88.6	91.4	72.8	58.3	85.1	97.0	71.2	71.1
EDPNet-c5	‘Xception+’+P1+AVP	78.4	110.0	507.7	89.1	91.4	71.1	64.4	87.4	97.0	71.3	71.9
EDPNet-c6	‘Xception+’+P1236 +ADAVP	77.9	107.0	507.7	87.8	90.6	68.7	65.5	88.3	96.8	71.5	70.6
EDPNet-c7	‘Xception+’+P1236 +MAX	78.4	107.0	507.7	88.7	91.7	70.8	62.1	87.4	97.1	74.7	71.5
EDPNet-c8	‘Xception+’+ P1236+AVP	77.2	107.0	507.7	88.2	91.6	70.7	60.4	85.9	97.0	72.2	70.1
EDPNet-c9	‘Xception+’+P1236 +ADAVP+DC	80.2	125.0	517.3	90.1	91.2	76.5	66.1	89.2	96.6	73.3	77.8
EDPNet-c10	‘Xception+’+P1236 +MAX+DC	79.7	127.0	517.3	89.7	90.7	78.9	64.3	86.3	96.2	72.2	77.5
EDPNet-c11	‘Xception+’+P1236 +AVP+DC	79.1	125.0	517.3	89.7	90.0	72.2	64.6	86.9	96.8	73.6	78.5

**Table 3:** Semantic accuracy of PSPNet, EDPNet-c6, and EDPNet with 8-fold downsampling on the eTRIMS validation images.

Models	mIoU (%)	Time (min)	Background	Building	Car	Door	Pavement	Road	Sky	Vegetation	Window
PSPNet [41]	83.0	174.0	66.8	92.4	93.0	75.6	70.5	92.6	98.0	73.7	84.2
EDPNet-c6	84.7	172.0	68.5	93.4	95.6	86.6	66.4	86.1	98.2	80.3	85.9
EDPNet	85.4	180.0	69.6	93.3	95.0	85.2	73.5	90.6	97.8	78.4	85.2

**Table 4:** Quantitative evaluation results of different models on the eTRIMS dataset. Note that all mIoU values are predicted based on eTRIMS test images.

Models	mIoU (%)	Time (min)	Background	Building	Car	Door	Pavement	Road	Sky	Vegetation	Window
FCN-8s [47]	56.6	120.0	21.1	81.9	61.6	34.6	27.6	67.2	93.7	64.4	56.7
LR-ASPP [64]	59.3	29.0	24.1	84.4	57.9	46.3	34.3	62.2	96.7	65.0	62.7
U-Net [50]	63.9	105.0	56.4	85.2	82.4	47.2	28.5	44.8	96.1	72.1	63.9
DeepLabv3 [63]	66.4	204.0	48.9	83.3	65.0	53.0	42.6	76.7	96.3	63.0	69.2
PSPNet [41]	83.0	174.0	66.8	92.4	93.0	75.6	70.5	92.6	98.0	73.7	84.2
HRNet [46]	77.7	100.0	62.4	93.4	90.7	63.6	57.6	85.9	97.4	78.2	70.2
EDPNet-c6	80.5	105.0	61.2	90.7	92.7	76.5	63.9	84.5	97.8	76.7	80.3
EDPNet	83.6	125.0	66.1	91.7	93.2	80.0	72.0	91.0	96.8	76.2	82.8

**Table 5:** Semantic segmentation accuracy of different models on the Cityscapes dataset.

Models	mIoU (%)	Time (h)	Road	Sidewalk	Building	Wall	Fence	Pole	Light	Sign	Vegetation	Terrain	Sky	Person	Rider	Car	Trunk	Bus	Train	Motorcycle	Bicycle
FCN-8s [47]	33.3	37.0	91.5	50.9	75.6	12.2	10.8	15.2	/	19.7	77.4	36.6	84.4	35.1	/	72.5	/	10.1	/	/	31.5
LR-ASPP [64]	36.6	16.0	92.2	53.9	78.3	22.9	13.6	18.4	/	22.1	80.7	43.1	85.4	38.7	/	75.8	/	16.2	/	/	34.3
U-Net [50]	53.5	47.0	95.2	66.7	84.9	32.0	32.0	37.5	32.0	46.4	85.9	47.6	90.9	57.9	31.8	84.9	45.2	44.7	22.8	23.4	54.3
DeepLabv3 [63]	42.0	102.0	93.8	62.0	82.0	24.6	25.5	26.9	10.5	31.8	85.2	43.0	88.4	47.1	/	80.4	10.3	27.9	12.4	/	43.8
PSPNet [41]	76.0	102.0	98.1	85.4	92.7	42.2	58.4	66.4	72.7	80.4	92.5	63.6	94.7	83.7	65.1	95.2	75.3	83.2	55.0	60.2	78.6
HRNet [46]	65.3	50.0	97.4	79.3	89.6	52.9	45.6	47.4	42.3	58.1	90.1	57.8	93.1	68.8	42.3	91.0	61.6	66.2	52.2	42.2	63.7
EDPNet-c6	75.6	53.5	98.2	84.7	92.6	58.9	64.3	60.8	70.3	78.9	92.3	60.7	94.3	82.2	65.3	95.4	82.8	78.2	28.2	69.9	78.6
EDPNet	71.2	60.0	97.6	82.4	92.3	50.1	54.6	59.0	68.5	77.1	91.7	61.0	93.8	80.3	62.3	92.9	42.8	59.5	53.1	56.5	76.5

The symbol “/” indicates that the IoUs of the corresponding class are less than 10%.

**Table 6:** Experimental results of different models on the PASCAL VOC 2012 dataset.

Models	mIoU (%)	Time (h)	Background	Aero	Bicycle	Bird	Boat	Bottle	Bus	Car	Cat	Chair	Cow	Diningtable	Dog	Horse	Motorbike	Person	Potted Plant	Sheep	Sofa	Train	TV
FCN-8s [47]	34.1	63.0	82.1	46.0	21.2	15.6	25.1	19.5	69.5	52.9	40.2	11.0	26.9	27.5	31.4	18.9	41.3	57.8	11.2	25.6	16.1	43.9	32.4
LR-ASPP [64]	36.1	15.4	82.3	52.0	22.6	27.6	31.5	23.9	58.0	55.7	38.2	11.3	30.9	29.0	33.6	18.5	46.8	55.0	11.5	30.6	18.0	47.9	33.8
DeepLabv3 [63]	37.1	123.5	83.2	60.2	20.5	24.0	38.9	21.9	68.8	55.2	39.3	12.9	27.6	23.3	26.2	24.7	51.9	61.5	16.1	31.1	15.2	53.5	22.9
U-Net [50]	55.9	131.5	90.0	70.2	33.1	66.0	51.2	49.9	71.7	69.1	72.2	22.5	51.6	33.4	61.3	52.7	62.4	73.5	40.3	55.3	28.9	64.3	54.6
PSPNet [41]	69.5	160.5	93.0	88.7	44.1	79.7	60.3	47.0	87.2	85.9	88.2	30.7	77.5	44.7	84.0	75.6	83.3	84.7	47.6	73.5	41.3	79.8	62.7
HRNet [46]	73.4	125.0	93.0	89.0	43.2	87.4	61.5	80.8	92.3	84.8	87.7	38.3	79.6	42.5	80.3	78.6	87.0	84.6	60.6	83.1.	43.3	81.0	62.6
EDPNet-c6	68.3	70.4	92.7	83.5	41.2	76.1	50.6	59.6	86.6	84.9	87.1	30.0	64.7	51.1	73.8	76.1	83.1	82.9	46.4	75.1	44.1	77.5	67.2
EDPNet	73.8	87.3	93.4	81.9	40.7	85.2	61.9	67.0	91.4	83.3	89.4	41.3	81.7	55.8	80.7	85.5	78.3	85.9	59.2	83.2	48.4	80.1	76.1

**Table 7:** The statistics of semantic accuracy of different models on the CamVid test dataset.

Models	mIoU (%)	Time (h)	Bicyclist	Building	Car	Fence	Pedestrian	Road	Sidewalk	Sky	Tree	Truck_bus	Vegetation Misc	Wall
FCN-8s [47]	51.1	16.0	34.1	77.6	58.8	31.8	6.8	85.6	65.7	87.9	71.3	18.9	31.2	44.0
LR-ASPP [64]	66.6	4.0	53.1	86.0	78.3	53,6	35.5	87.6	68.4	89.8	77.5	76.5	47.3	46.1
U-Net [50]	74.4	21.0	75.4	88.2	79.2	71.1	48.4	93.9	84.6	92.6	81.1	79.2	61.5	66.2
DeepLabv3 [63]	65.1	26.0	63.6	87.6	77.1	49.9	39.9	91.5	84.1	90.9	81.1	77.6	49.8	52.5
PSPNet [41]	82.6	27.5	82.6	92.5	91.0	83.3	69.0	95.0	89.1	91.6	82.2	90.5	70.2	76.8
HRNet [46]	74.0	22.0	75.8	88.8	88.4	60.5	51.9	93.7	82.5	91.2	79.3	77.6	51.8	47.0
EDPNet-c6	79.4	12.9	76.6	92.0	90.4	81.1	58.8	93.5	87.4	91.0	81.7	86.9	66.6	75.4
EDPNet	80.8	15.2	79.5	92.2	91.0	82.3	60.8	94.1	88.8	91.1	81.8	88.9	68.1	76.5

## Data Availability

The experimental datasets of eTRIMS, Cityscapes, CamVid, and PASCAL VOC 2012 presented in this paper are available on request from the corresponding benchmark websites or distribution platforms. The source code of the EDPNet has been made available at https://github.com/EDPNet/EDPNet, accessed on 19 February 2023.
